# Source, Spatial Distribution and Pollution Assessment of Pb, Zn, Cu, and Pb, Isotopes in urban soils of Ahvaz City, a semi-arid metropolis in southwest Iran

**DOI:** 10.1038/s41598-019-41787-w

**Published:** 2019-03-29

**Authors:** Ahad Nazarpour, Michael J. Watts, Ayoub Madhani, Somayeh Elahi

**Affiliations:** 1 Department of Geology, Ahvaz Branch, Islamic Azad University, Ahvaz, Iran; 2 Inorganic Geochemistry, Centre for Environmental Geochemistry, British Geological Survey Keyworth, Nottingham, UK; 3 Department of Chemistry, Abadan Branch, Islamic Azad university, Abadan, Iran; 4 Department of civil engineering, Abadan Branch, Islamic Azad University, Abadan, Iran

## Abstract

This study examined the status of toxic metal contamination of the urban industrial city of Ahvaz in Iran. Two hundred and twenty-seven surface soils from a depth horizon of 0–10 cm were collected from urban areas. In addition, 15 soil samples were collected to recognise the sources of Pb in urban topsoils in Ahvaz city. Mean concentration of Pb, Zn, Cu and As were 181 ± 167, 123 ± 118, 185 ± 167 and 6.9 ± 8.9 mg.kg^−1^, respectively. Results of inter-element relationship among studied toxic metals revealed that Pb, Zn and Cu may have the same anthropogenic origin, whilst As originated from different sources. The results of pollution index (PI) and Nemerow pollution index (NPI) implied that Pb, Zn, and Cu had a moderate to high level of pollution. The Pb isotopic composition analysis suggested clear anthropogenic origins of Pb including industrial emission, vehicle exhaust and dust storm with the mean contributions of 47%, 15% and 7%, respectively, by a four-end member model.

## Introduction

With the rapid growth and industrial improvement in recent decades, urban environments are increasingly determining human health and wellbeing^1^. Urban surface soil as an attribute of urban environments is the primary sink of potentially toxic metals and other pollutants^2–4^. In general, subsurface and surface soil contains natural quantities of potentially toxic metals, so-called baselines. The baseline concentrations depending on the nature and constitution of parent rock material^5–7^. Amongst different pollutants, potentially toxic metals are harmful to public health and the urban ecosystem^8,9^. Humans are exposed to potentially toxic metals contained in soil via three main pathways, including inhalation, ingestion and skin exposure^4,10–14^. Potentially toxic metals can have adverse impacts on the central nervous system, cardiovascular, and bones^10^. Children are particularly at risk to potentially toxic metal pollution owing to rapid mental and physical development^15^. The anthropogenic origins of potentially toxic metals including lead (Pb), zinc (Zn), copper (Cu), and arsenic (As) are mainly attributed to traffic, vehicle emission, brake and tyre wear and street industrial activities. In addition, industrial discharge of potentially toxic metals into the urban environment can include: power and desalination plants, oil well drilling activities, fuel combustion, local industrial zones, metallurgical industry, household release, weathering of asphalt and roadside material, atmospheric and dust deposition^16–23^. The common applied toxic metal pollution indices in soils and sediments can be defined in two main categories: single and integrated indices^24–26^. Firstly, single methods include the enrichment factor (EF), index of geo-accumulation (Igeo) and pollution index (PI), which which provide a measure of single metals and distinguish background from threshold pollution level^26,27^. Secondly, integrated indices such as the integrated pollution index (IPI), Nemerow pollution index (NPI), and risk index (RI) are applied to more than one metal and is an integration of toxic metal pollution values for every sampling point and can be composed by each of the single indices^27,28^.

The measurement of total Pb in environmental samples provides valuable information regarding the level of contamination^16,29^. However, it does not provide an estimation of the origin of Pb. Usage of Pb isotopes can clear the origin of Pb contamination and differentiate the potential anthropogenic source of Pb from geogenic sources of Pb^30–33^. The main naturally occuring of lead isotopes are: ^204^Pb, ^206^Pb, ^207^Pb, and ^208^Pb. While ^204^Pb is the only non-radiogenic isotope and thus it’s concentration in soil is constant over time^31^, whilst ^206^ Pb, ^207^ Pb, and ^208^ Pb are ^238^U, ^235^U, and ^232^Th end members of the decay series, respectively. Since the Pb isotopic ratios are not considerably influenced through physico-chemical fractionation processes such as smelting, purifying, manufacturing, and industrial activities^29^, different or overlapping isotopic ratios of Pb can be observed by integration of natural and anthropogenic sources of Pb^34^.

Ahvaz as a major industrialised center is one of the fastest developing metropolises in Iran. In this current study, we present the first comprehensive report on the spatial distribution, pollution level and source identification of main anthropogenic sources of toxic metals (Pb, Zn, Cu and As) by a systematic sampling strategy in urban topsoils in Ahvaz City. Previous reports have applied a geographical information sysytem (GIS) survey to study the distribution and hot-spot identification of potentially toxic metal pollution assesment in urban soils^35–37^. However, GIS-based work in soil environmental quality has never been reported in Ahvaz. The current study aims are: (1) to present the spatial distributions of potentially toxic metals in surface soils in Ahvaz city; (2) to identify the co-sources of pollutants with the application of robust multivariate analysis (principle component) and Pb isotopic signature; (3) to explore the degree of toxic metal pollution in the soils by applying pollution indices to provide a metric for hazard to human health; (4) application of Pb isotope tracing method to identify the source of metal pollution in surface soils.

## Study Area

**Figure 1:**
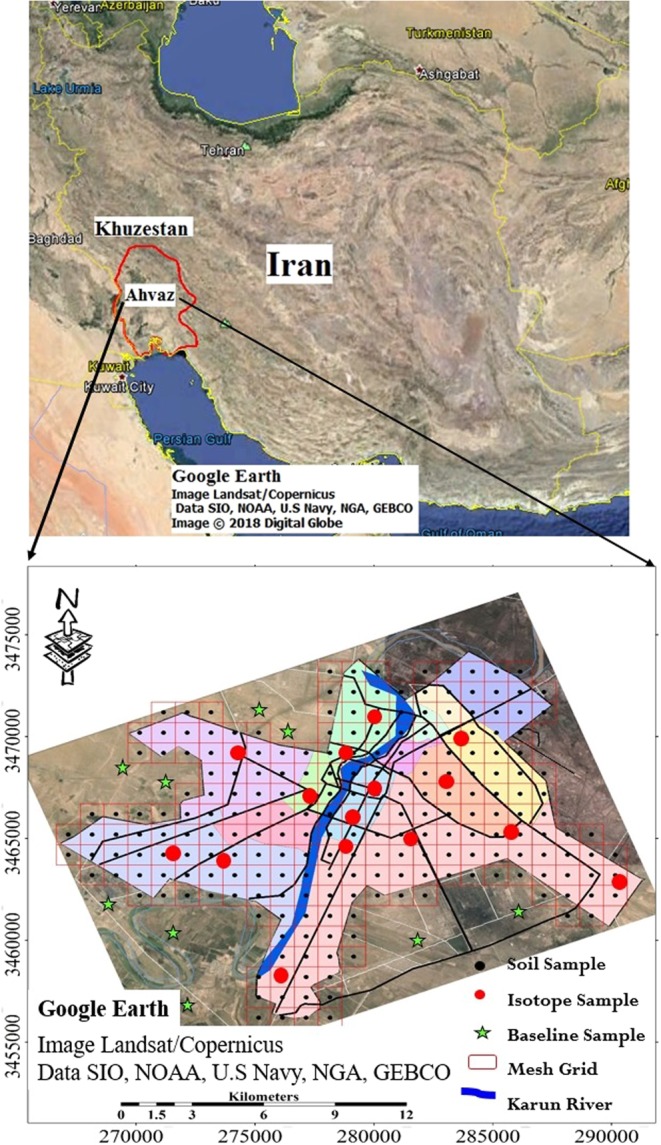
(**A**) Location of Khuzestan province and study area in the southeastern part of Iran, (**B**) sampling sites, the image was made by ArcGIS10.2, background from Google Earth (Image: Google, Landsat/Copernicus).

Ahvaz city as the capital of Khuzestan province, is situated in 31° 20*N*, 48° 40*E* 12 meters above sea level in the south west of Iran (Fig. 1) with 1.5 million inhabitants. It is situated in an arid area near Iraq, Kuwait, Syria, and Saudi Arabia, which are the major origins of sand and dust storm events in the Middle East. In addition, Ahvaz city is located on the Ahvaz oil field, one of the most important Iranian giant oil fields, with more than 450 active wells. The existence of great industrial hubs, i.e., Iran National Steel Industrial Group (INSIG), Khuzestan Steel Company (KSC), National Iranian Drilling Company (NIDC), Carbon Black (CB) company, local industrial zones, pipe industry, official and industrial facilities and a transportation junction from the Persian Gulf bays from the southwest to all parts of the country, has turned Ahvaz into one of the most important industrial, economic, educational, cultural, manufacturing and high-tech industrial centres in Iran.

The average temperature in Ahvaz city is 32 °C in January, 38 °C in April and 49 °C in July. Average of annual rainfall is 213 mm per year, mainly falling December to April. The study area is characterised as mainly alluvial and sedimentary rocks, with sand and silt fractions comprised of quartz, and fine silt and clay fractions mainly controlled by clay minerals^38^.

Soils in this study were categorized as sandy loam to loam, and clay loam^39^. The pH of these soils was determined in CaCl_2_ 0.01 M solution and ranged from 7.9 to 8.2, that suggest sub-alkaline property for topsoils in the study area^40,41^.

## Material and Methods

Two hundred twenty-seven (227) topsoil samples from (0–10 cm) were collected in July 2015 (dry season), to prevent rain/flood washing out the potentially toxic metals. A regular grid sampling plan was implemented to specify a systematic sampling system (240 km^2^). The study area was divided into 227 grid nodes of 1 km^2^. Each soil sample was carefully obtained from a composite of five sub-samples (0–10 cm), with a plastic trowel and mixed samples sieved through a 2 mm nylon mesh, air-dried and stored in polyethylene bags. Deionised water was used to clean the trowel before and after sample collection. Sampling sites are displayed in Fig. (1). In addition, eight sub-surface soil samples as a baseline were collected from eight pits at a depth of 120 cm.

To characterise metal concentrations of soil samples (<63 μm fraction) were digested using HNO_3_ (65%) + HCl (37%) + HF (40%) as stated in the EPA 3050B1 method^42^. Digested samples were passed through an 8μm membrane filter and diluted with Milli-Q water. Selected toxic metals (Pb, Zn, Cu and As) were measured by flame atomic absorption spectrophotometry (PG990 Model). Quality control and quality assessment (QC/QA) included analytical duplicates samples, a reagent blank, standard reference materials (SRM- Montana I SRM-2710) (multi-element soil standard OREAS45EA and OREAS24P) and procedural blanks provided a measure of analytical performance. The average of recovery values ± SD in SRMs for selected metals were Pb (99 ± 3.23, Zn (102 ± 10.25), Cu (99 ± 4.58) and As (99 ± 4.87), which indicating a good agreement between measured and certified value. Detection limits for selected metals in AAS was calculated by using 3 × SD (standard deviation) of concentration of ten blank samples. Detection limit were 0.05 mg.kg^−1^, 0.04 mg.kg^−1^, 0.02 mg.kg^−1^, and 0.03 mg.kg^−1^ for Pb, Zn, Cu and As, respectively. Precision, specified by duplicate measurements was ±5% for all selected metals.

Fifteen topsoil samples and four samples used as ‘control’ soils from areas with low traffic density with no industrial activities at a depth of 50 cm were collected and Pb isotopic ratios ^204^Pb, ^206^Pb, ^207^Pb, and ^208^Pb determined in samples along with SRM-981 (National Institute of Standards and Technology; NIST, USA) by Inductively Coupled Plasma-Mass Spectrometry (ICP-MS; Perkin-Elmer Elan 6100 DRC^plus^). Comprehensive analytic specifications of Pb isotope quantities are provided in Ettler, *et al*.^43^ and MacKenzie and Pulford^44^. Lead isotopes ratios including ^204^Pb,^206^Pb, ^207^Pb, ^208^Pb that are used in current study have been frequently interpreted in previous studies^45–47^. Correction for mass bias during the determination of the isotopic ratios was performed using analyses of NIST SRM-981 after every three samples. The measured values for NIST SRM-981 were ^206^Pb/^204^Pb = 15.9773 ± 0.0054, ^207^Pb/^204^Pb = 15. 3536 ± 0.0063, ^208^Pb/^204^Pb = 35.6784 ± 0.0175, ^207^Pb/^206^Pb = 0.894131 ± 0.0181, and ^208^Pb/^206^Pb = 2.15342 ± 0.0123 (2σ, external standard deviation, n = 5). The standard errors for measurement of the ^206^Pb/^207^Pb and ^208^Pb/^206^Pb ratios were <0.5% and <0.4% relative standard deviation (RSD), respectively.

### Descriptive and statistical analysis

To assess the relationship among variables, correlation coefficient, multivariate analysis including robust principle component analysis (RPCA) was applied using R packages (see http://cran.r-project.org/). PCA is extensively applied to decrease data dimension and to extract relationship among the experimental variables. The PCA method involves an unsupervised classification process that includes determining the geochemical association based on correlation anlysis or similarity among variables to be classified according to their source similarity^48^.

## Potentially Toxic Metal Pollution Assessment

Pollution index (PI) is a powerful tool for processing, analysing, and conveying raw environmental information to decision makers, managers, technicians, and the public^49^. The pollution index (PI) for every studied toxic metal and the Nemerow pollution index (NPI) for all studied toxic metals were calculated to assess the potential hazard associated with the soil samples. The PI is calculated as:

1 $$\begin{array}{ll}PI={{\rm{C}}}_{{\rm{i}}}/{{\rm{X}}}_{{\rm{a}}} & {{\rm{C}}}_{{\rm{i}}}\le {{\rm{X}}}_{{\rm{a}}}\\ PI=1+\,({\rm{X}}-{{\rm{X}}}_{{\rm{a}}})/({{\rm{X}}}_{{{\rm{C}}}_{{\rm{i}}}}-{{\rm{X}}}_{{\rm{a}}}) & {{\rm{X}}}_{{\rm{a}}} < {{\rm{C}}}_{{\rm{i}}}\le \,{{\rm{X}}}_{{{\rm{C}}}_{{\rm{i}}}}\\ PI=2+(X-\,{{\rm{X}}}_{{{\rm{C}}}_{{\rm{i}}}})/({{\rm{X}}}_{{\rm{p}}}-{{\rm{X}}}_{{{\rm{C}}}_{{\rm{i}}}}) & {{\rm{X}}}_{{{\rm{C}}}_{{\rm{i}}}} < {{\rm{C}}}_{{\rm{i}}}\le {{\rm{X}}}_{{\rm{p}}}\\ PI=3+(X-{{\rm{X}}}_{{\rm{p}}})/({{\rm{X}}}_{{\rm{p}}}-{{\rm{X}}}_{{{\rm{C}}}_{{\rm{i}}}}) & {{\rm{C}}}_{{\rm{i}}} > {{\rm{X}}}_{{\rm{P}}}\end{array}$$

where C_*i*_ is concentration of each toxic metal *i*, and X_a_, X_c_ and X_p_ are threshold concentrations of toxic metal indicating enrichment, low pollution intensity, and high pollution intensity, respectively. Methods for estimations of X_a_, X_c_ and X_p_ are explained in Table (1). Then, the PI was categorized as follow: non-polluted for PI < 1, low pollution level (1 < PI < 2), moderate level of pollution (2 < PI < 3), and high pollution level (PI > 3)^4^:

**Table 1:** Threshold concentrations of each level of pollution for each metal (mg.kg^−1^).

	X_a_	X_c_	X_p_
As	15	25	30
Pb	35	250	500
Cu	30	50	400
Zn	85	200	500

The NPI of every sample i was calculated as^4^:

2 $$NPI=\sqrt{\frac{P{I}_{i\,max}^{2}+P{I}_{i\,ave}^{2}}{2}\,}$$

where PI_imax_ and PI_iave_ are the maximum and average values of PI for every metal, respectively^4^, NPI values classify as: non-pollution (NPI ≤ 0.7); warning line of pollution (0.7 < NPI ≤ 1); low pollution level (1 < NPI ≤ 2); moderate pollution level (2 < NPI ≤ 3) and high pollution level (NPI > 3).

## Results and Discussion

### Potentially toxic metal concentration

Results of analytical data indicated that the coefficient of skewness for Pb, Zn, Cu and As were extensively higher than zero, showing a right skewed distribution (Table 2). It shows that samples with high value of Pb, Zn, and Cu occurred in the collected samples and indicated the non-similar distribution of concentration values. In contrast, As with close to zero skewness coefficients demonstrate a normal distributions. High standard deviation (SD) were observed in all potentially toxic metals except As, revealing the large variation of toxic metals in Ahvaz topsoils. The mean values of Pb, Zn, Cu and As in Ahvaz topsoils were noticeably greater than the corresponding baseline concentrations, demonstrating the pollution from anthropogenic sources and considerable contamination level in the Ahvaz city. Findings achieved for studied toxic metals are reviewed in the following sections:

**Table 2:** Descriptive statistics of the metal concentrations determined in urban soil samples, and in soil samples from non-human impacted soils in the urban fringe (mg.kg^−1^).

Metal	Minimum (mg kg^−1^)	Maximum (mg kg^−1^)	Average (mg kg^−1^)	Std. Deviation	Variance	Skewness	Mean standard deviation of baseline value (mg kg^−1^)
Pb	9.36	793.3	181	167	2815	1.25	12.5 ± 2.3
Zn	13	297	123	118	334	0.1	15.7 ± 3.4
Cu	8	1060	185	167	273	1.7	29.5 ± 3.7
As	0.5	18.3	6.9	8.9	29	0.02	3.5 ± 1.1

### Pb

Total Pb concentrations had a considerable range from 9.36 to 793.3 mg.kg^−1^ with an average value of 181±167 mg.kg^−1^, with 97% of topsoils higher than the baseline value of 12.5 ± 2.3 mg.kg^−1^. The average value of 181 mg.kg^−1^ Pb in Ahvaz surface soil is higher than the target value of 85 mg.kg^−1^ suggested by The Netherlands soil contamination guideline^50^; greater than the 130 mg.kg^−1^ mean Pb found for 34 European cities^51^; 35 mg.kg^−1^ Pb in 21 Chinese cities^51^; 112 mg.kg^−1^ Pb in Sialkot^52^; and lower than 231 mg.kg^−1^ in Baltimore^53^; 395 mg.kg^−1^ in Chicago^54^ in the USA; 208 mg.kg^−1^ in Islamabad^55^, Pakistan, and 262 mg.kg^−1^ in Naples, Italy^29^ (Table 3).

**Table 3:** Comparison of mean concentration (mg.kg^−1^) of metal in urban soils from different cities.

City	As	Cu	Pb	Zn	Reference
34 European Cities	13	46	102	130	^51^
Baltimore (USA)	—	45	231	141	^53^
Chicago (USA)	20	150	395	397	^54^
Mexico (Mexico)	—	101	140	307	^14^
Ibadan (Nigeria)	3	32	47	94	^60^
Bangkok (Thailand)	—	42	48	118	^61^
Islamabad (Pakistan)	74	18	208	1643	^55^
Izmit (Turkey)	—	37	35	72	^59^
Sialkot (Pakistan)	—	19	112	72	^52^
21 Chinese cities	12	30	35	90	^51^
Ahvaz (Iran)	6.9	185	181	123	This Study

It is probable that in newly developed districts, the total amount of Pb in soils were less than 20 mg.kg^−1^ but in places with high residential and historical general, low level concentrations have elevated values from 30–100 mg.kg^−1 5^. In this investigation, 44% of the soil samples had Pb values greater 100 mg.kg^−1^, these samples were located in areas with population density, high traffic volume, and presence of industrial hubs such as drilling activities (mostly oil-based mud pits), pipe industry, Black Carbon company (BC), Iran National Steel Industrial Group (INSIG), Khouzestan Oxin Steel Company (KOSC), Khuzestan Steel Company (KSC) and local industrial zones. Therefore, locations with high contamination of Pb, suggesting that human activities are the major sources of Pb in the topsoil of Ahvaz city.

### Zn

Even though Zn is an essential micronutrient for a healthy body, extreme amounts of zinc can be destructive, and cause Zn toxicity^56,57^. High levels of Zn can interrupt the balance of other essential minerals to sustain healthy life, such as Fe and Cu^10^. Anthropogenic Zn is common in car lubricants, tires and Carburettors^58^.

The range of Zn value was 13–297 mg.kg^−1^, with an average of 123 ± 118 mg.kg^−1^, which is greater than the average concentration of baseline samples with 15.7 ± 3.4 mg.kg^−1^. The average value of Zn in Ahvaz surface soil is also greater than 72 mg.kg^−1^ in Izmit^59^ and Sialkot^52^, 94 mg.kg^−1^ in Ibadan^60^, 90 mg.kg^−1^ in 21 Chinese cities^51^, but it is lower than 130 mg.kg^−1^ in 34 European Cities^51,53^ 141 mg.kg^−1^ Baltimore^53^, 397 mg.kg^−1^ in Chicago, 1643 mg.kg^−1^ in Islamabad, and 118 mg.kg^−1^ in Bangkok^61^ (Table 3).

### Cu

The concentration of Cu in soil samples of Ahvaz ranged from 8–1060 mg.kg^−1^, with an average of 185±167 mg.kg^−1^. This concentration is noticeably higher than the Ahvaz baseline soil-Cu concentration of 29.5± 3.7 mg.kg^−1^, however, the mean Cu value is markedly greater than 46 mg.kg^−1^ found in 34 European cities^51^, the 45 mg.kg^−1^ in Baltimore^53^, the 150 mg.kg^−1^ in Chicago^54^ in the USA, the 101 mg.kg^−1^ in Mexico^14^, the 47 mg.kg^−1^ in Naples^29^ and 63 mg.kg^−1^ in Palermo^62^ (Table 3).

### As

The As concentrations with a range of 0.5–18.3 mg.kg^−1^ have an average of 6.9 ± 8.9 mg.kg^−1^ (Table 2). The mean value of As in the baseline soil is 3.5 ± 1.1 mg.kg^−1^. As shown in Table 2, the mean value of the As in the Ahvaz urban soil samples is much lower than those stated from many large and/or industrialised cities as reported in Table (3).

**Figure 2:**
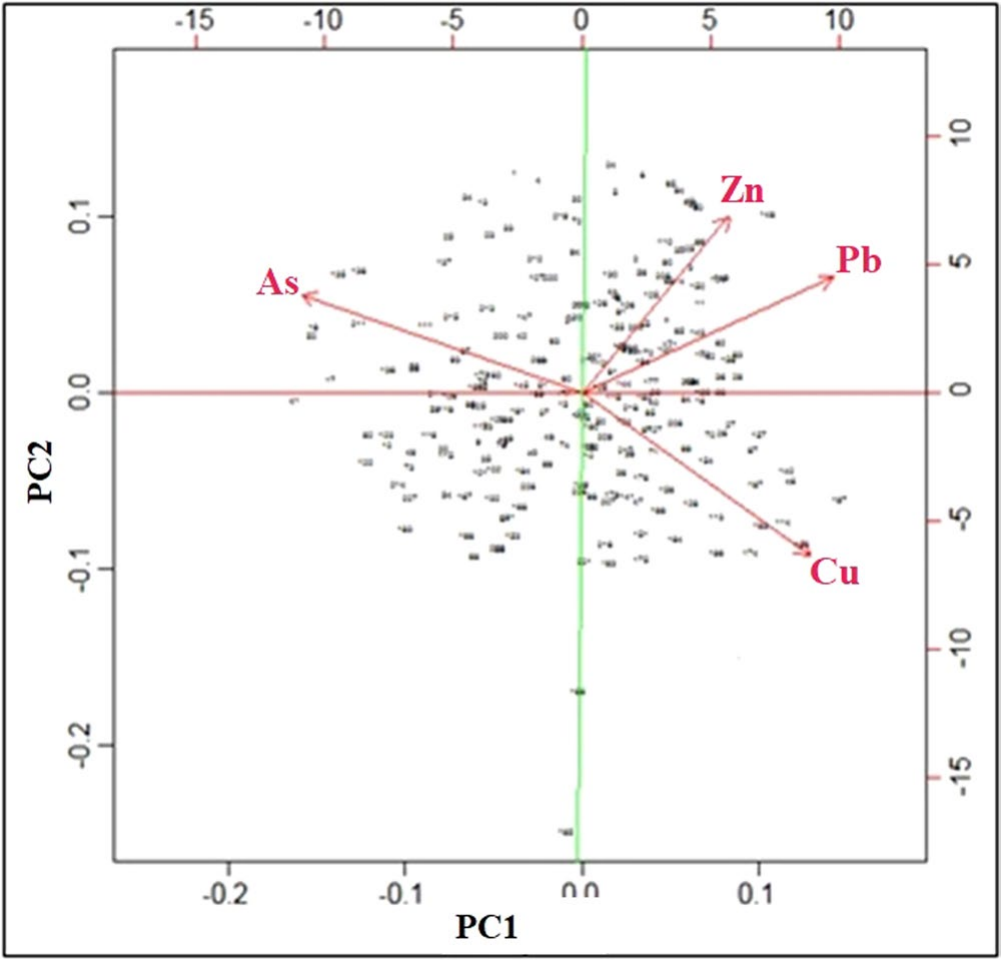
Biplots of the first and second PCs.

PCA has been performed to further identify the source of potentially toxic metals^63,64^. PC1 with 82% of total variance was the most important component and high loadings dominated by Pb (0.723), Zn (0.602) and Cu (0.874) had a significant positive correlation. PC2 with 11% with variance loading was dominated by As (0.641) seperated from other toxic metals thay may indicate different sources or different geochemical behavior of As^65^. In addition, the correlation of PC1 and PC2 achieved from the biplot (Fig. 2) indicates that the PC1 scores show a positive relationship between Pb, Zn and Cu, with eigen values greater than 0.6, confirm that these metals originate from common sources, markedly emission sources as reported in earlier studies^21,66,67^. Traffic related pollutants are mainly vehicle exhaust and other particles form tyre and brake lining erosion^68^. As previously reported, the major source of Pb is the fuel combustion of leaded gasoline^69,70^. Motor lubricating oil leakage, street paint, mechanical abrasion, car parts wearing-out, corrosion, physical abrasion of vehicles can also be considered as the sources of Zn in urban soil^71–73^.

It appears that PC1 metals are from anthropogenic origins, while the second factor with a positive loading of As might be from natural origins and resuspension of soil-derived particles^74,75^. As shown, there is a relatively weak association with other potentially toxic metals. On the other hand, the As concentration in urban soils was larger than those of baseline areas. Therefore, there seems to be an additional As source to the geogenic one, although this source is apparently different from the other metals.

### Metal pollution assessment

The pollution index (PI) was evaluated using the baseline values of potentially toxic metals in the urban surface soils of Ahvaz. Pollution index (PI) ranges for Pb were from 0.3 to 4.2 with an average value of 2.3. Approximately, 68% of the analysed soil samples indicating moderate to high PI values for Pb. The PI value for Cu and Zn represent moderate to high levels of pollution, with a range of 0.3 and 5.9 for Cu and 0.15 and 2.3 for Zn. The moderate to high PI values were obtained in 34 and 32% of soil samples for Cu and Zn, respectively.

**Figure 3:**
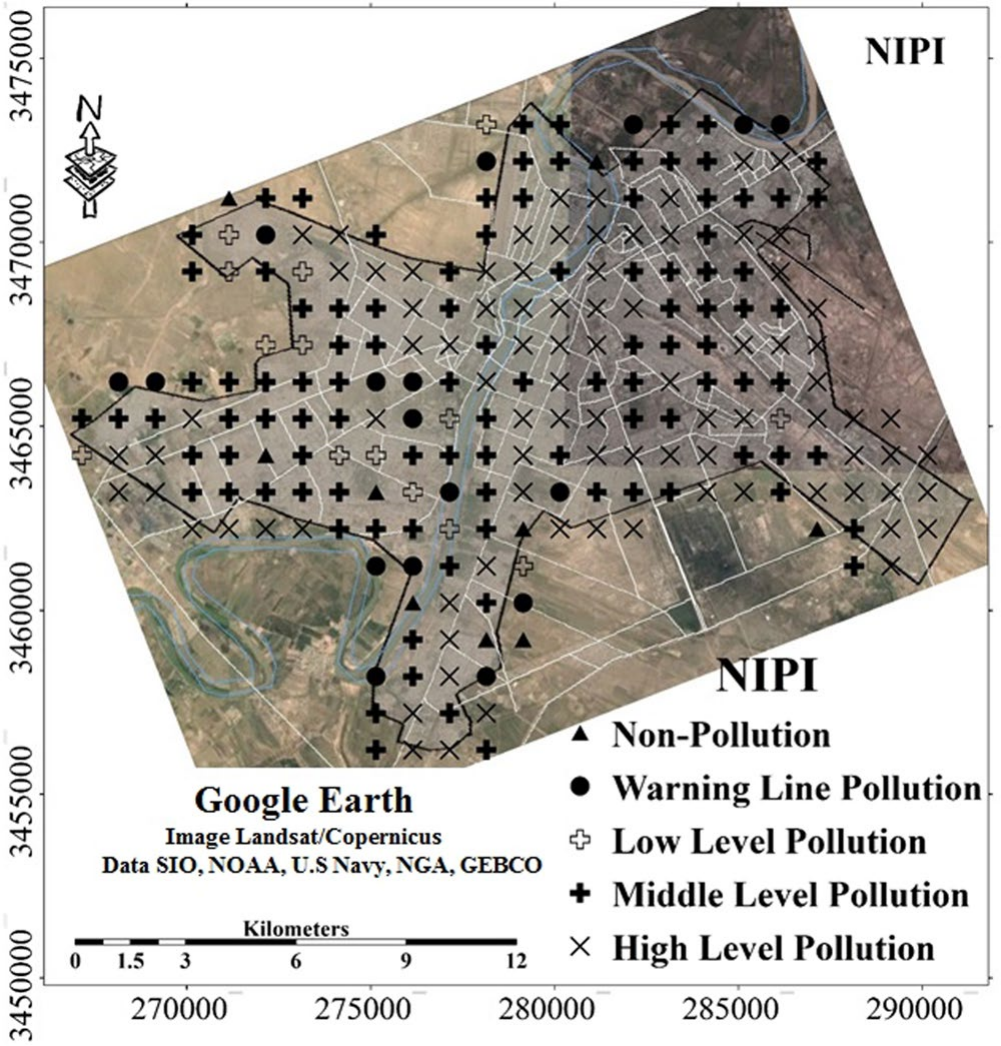
Spatial distribution of the Nemerow pollution index (NPI) in the studied area, the image was made by ArcGIS10.2, background from Google Earth (Image: Google, Landsat/Copernicus).

The NPI values ranged from 0.3 to 4.5 with a mean value of 2.8. Figure (3) shows the spatial distribution map of NPI in Ahvaz soils. The evaluation of the analysed samples indicates that the surface soil of Ahvaz city have noticeably been effected by potentially toxic metals. About 38% of soil samples had high levels of pollution with a NPI > 3. There is an obvious distribution in the spatial distribution map of PI and NPI in Ahvaz city that highlights a rapid urbanisation with no defined residential and industrial zones.

Furthermore, evaluation of the data indicated that only nine samples (4% of all soil samples) had an NPI < 0.7, which were classified as non-pollution. Approximately 8% and 6% of all soil samples were on the NPI warning line and suggested low-level pollution, respectively, whilst 44% and 38% of all samples had an NPI between 2 and 3 and NPI ≥ 3, respectively, which indicates moderate and high levels of pollution.

### Isotope composition

**Figure 4:**
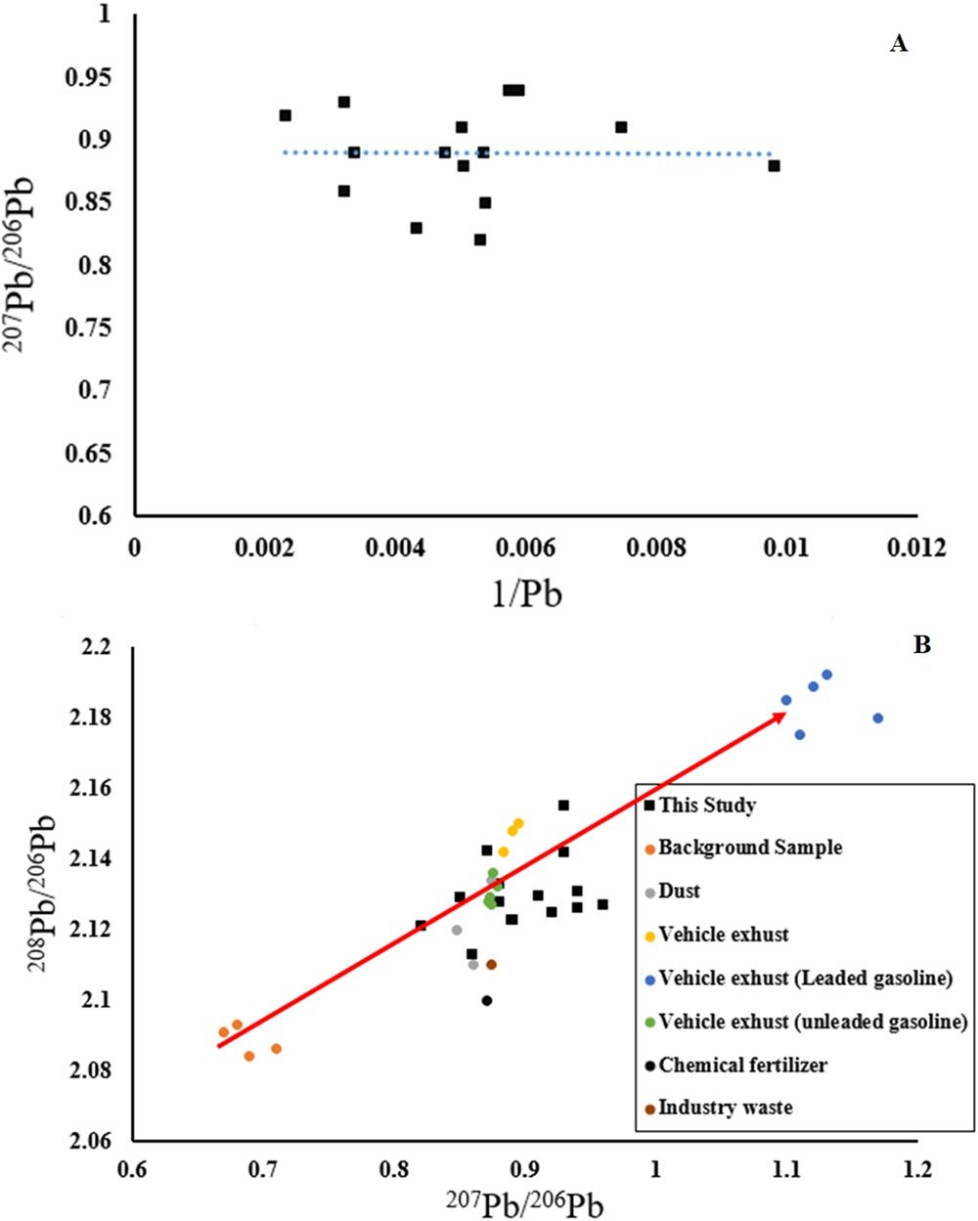
Plots of ^207^Pb/^206^Pb versus 1/Pb (**A**) and ^208^Pb/^206^Pb versus ^206^Pb/^207^Pb (**B**) in Ahvaz urban soil and the known sources, data of natural background, vehicle exhausts of leaded and unleaded gasoline, chemical fertilizer, dust are from references therein.

The range of ^206^Pb/^204^Pb, ^207^Pb/^204^Pb, ^208^Pb/^204^Pb were 18.4–17.3, 16.4–14.4, and 42.8–38.2, respectively. The highest average ratio of ^206^Pb/^204^Pb (19.05) indicated Pb in soils from fuel combustion sources^45–47^. A plot of 1/Pb and ^207^Pb/^206^ could be applied to identify the source of Pb in surface soils^46,76^. Results indicated non-significant linear relationship between 1/Pb and ^207^Pb/^206^ (R^2^ = 0.0076, Fig. (4A), which implying that combination of major anthropogenic emission as well as the geogenic sources in the Ahvaz surface soils^46,77^. In addition, plot of ^208^Pb/^206^Pb and ^208^Pb/^206^Pb of natural background and potential Pb pollution sources indicating a non-linear correlation in the selected soil samples proposing a combination of a complex constituents with distinct Pb isotopic ratios^70,78^. In addition, Fig. (4B) indicate that the isotopic composition of Pb revealing that industrial emission, vehicle exhaust emissions, dust deposition, and parent materials are the major resources of Pb in the Ahvaz surface soil samples. Even though, Pb isotopic ratios of vehicle exhaust were reasonably scattered as leaded and unleaded samples, the Pb isotopic ratios in studied soil samples were clearly in adjacent to the vehicle exhaust samples (Leaded and unleaded gasoline), industrial waste, and chemical fertilizer^47,79^ (Fig. 4B). Therefore, it can be concluded that vehicle exhaust/emission and dust deposition were the main contributors of Pb deposition in the soil environment. According to the above results, we can conclude that the Pb concentration in the studied soil samples could be the consequence of natural sources including geogenic process and anthropogenic activities.

In addition, in this investigation the Pb isotopic composition of Ahvaz dust collected during a storm of differing particulate matters was used to represent the isotopic composition of Ahvaz Pb isotopic ratios on dusty days^33^. The mean ^207^Pb/^206^ of Ahvaz soils (0.98) is higher than the natural baseline soils (0.68), and both of them are lower than the ^207^Pb/^206^Pb average value of Ahvaz dust samples in dusty days. The average of ^208^Pb/^206^Pb ratio of Ahvaz soil samples (2.122) is higher than the natural baseline value (2.088) and lower than the Ahvaz dust mean value (2.14). The higher ^208^Pb/Pb^206^ and ^207^Pb/^206^Pb ratios in Ahvaz topsoils compared to baseline samples suggest two distinct sources of Pb; one of obvious natural source (parent material/geogenic) and the other of anthropogenic contributors such as vehicle emission (leaded gasoline), industrial releases and atmospheric deposition^46,70,80^.

A nonlinear mixing of four-end-member technique was utilised to calculate the contribution of natural sources (f_1_), industrial emission (f_2_), vehicle emission (f_3_), and dust deposition (f_4_) to total Pb in soil samples^46,47,81^.

3 $$f1+f2+f3+f4=1$$

4 $$\frac{f1\times {C}_{s}}{{C}_{1}}+\frac{f2\times {C}_{s}}{{C}_{2}}+\frac{f3\times {C}_{s}}{{C}_{3}}+\frac{f4\times {C}_{s}}{{C}_{4}}=1$$

5 $$f1\times R1+f2\times R2+f3\times R3+f4\times R4={R}_{S}$$

where, C1, C2, C3, C4, are the average of Pb concentration of baseline soils (27 mg.kg^−1^), industrial release (6682 mg.kg^−1^), vehicle emission (2380 mg.kg^−1^), and surface dust as a function of upper crust concentration (17 mg.kg^−1^) respectively^82,83^.

The factors of *R1, R2, R3* and *R4* are the average values of ^206^Pb/^207^Pb ratios of natural background soil (1.21)^84,85^, industrial release (1.170)^46,82^, and vehicle exhaust (1.130)^46,47,86^, Ahvaz dust storm sample (0.96)^87^ respectively. Cs and Rs factors are Pb concentration and the ^206^Pb/^207^Pb value of every soil sample. The determined inputs of surface soil as a function of natural (geogenic) source, vehicle emission, industrial release, and dust source to total Pb in the each of soil samples are presented in Table (4). According to the integration end-member model the values of natural background, industrial release, vehicle emission and dust storm are 42–51%, 12–49%, 5–45% and 5–12%, respectively (Table 4). It is indicated that, in the Ahvaz city, industrial activities including oil well waste, not well defined land use planning companies, such as the Carbon Black Company, steel companies and local industrial zones are the main anthropogenic lead sources in surface soil of Ahvaz city.

**Table 4:** Percent of each possible sources of Pb in the surface soil samples of Ahvaz City.

Sample	Background/geogenic	Industrial emissions	Vehicle exhaust	Dust storm
1	28.5	49.12	15.9	6.48
2	36.5	32	31.5	0
3	28	46.3	19.5	6.2
4	24.8	37.2	22.2	15.8
5	25.1	40.8	17.7	16.4
6	32.4	46	13.6	8
7	34.3	53.2	12.5	0
8	33.2	47.39	19.41	0
9	34.5	43	14	8.5
10	28.9	61.1	6.5	3.5
11	30.4	48.2	12	9.4
12	30.8	41.6	20.5	7.1
13	31.3	44.3	19.1	5.3
14	35	53.5	0	11.5
15	27.2	47.7	17	8.1
**Mean**	30	47	15	7

## Conclusions and Remarks

In the present investigation, the total concentration of toxic metals in the Ahvaz surface soils was examined. Lead isotope ratios were analysed to identify the major source of Pb in the Ahvaz surface soil. Mean concentrations of Pb, Zn, Cu and As were higher their corresponding baseline levels. Coefficient correlation, principle component and cluster analysis indicated that distinct types of toxic metals from anthropogenic sources can be separated. Specifically, the As value which may be controlled by different sources from one of the other metals, whereas the level of Pb, Zn and Cu in Ahvaz soils mostly originate from common anthropogenic contaminations such as vehicle emissions and industrial sources. Pollution index values indicated that there was no significant pollution for As, but 68, 32 and 34% of the analysed urban soil samples were highly polluted with Pb, Zn and Cu, respectively. The NPI of the four potentially toxic metals also demonstrated that soil samples in Ahvaz show moderate to high levels of pollution. More than 44 and 38% of soil samples had a 2 < NPI < 3 and NPI ≥ 3, indicating moderate and high level pollution, particularly in the areas with high traffic volumes and industrial zones. Calculated four-end member model of Pb isotopic ratios suggested that industrial emission, vehicle exhaust and dust storm with the mean contributions of 47, 15 and 7% are the main source of Pb in Ahvaz surface soil.

## Supplementary information

Supplementary Dataset 1
