# Supplementary material for: Source, Spatial Distribution and Pollution Assessment of Pb, Zn, Cu, and Pb, Isotopes in urban soils of Ahvaz City, a semi-arid metropolis in southwest Iran
Source: Sci Rep. 2019 Mar 29;9:5349. doi: 10.1038/s41598-019-41787-w (PMC6441049; doi:10.1038/s41598-019-41787-w)
Supplement: Supplementary file 1 — Supplementary Dataset 1 [file 41598_2019_41787_MOESM1_ESM.pdf]

Source, Spatial Distribution and Pollution Assessment of Pb, Zn, Cu, and Pb, Isotopes in urban soils of Ahvaz City, a semi-arid metropolis in southwest Iran

Ahad Nazarpour[1✉], Michael J. Watts[2], Ayoub Madhani[3], Somayeh Elahi[3]

1- Department of Geology, Faculty of Science, Ahvaz Branch, Islamic Azad University, Ahvaz, Iran

2- Inorganic Geochemistry, Centre for Environmental Geochemistry, British Geological Survey Keyworth UK

3- Department of Chemsitry, Abadan Branch, Islamic Azad university, Abadan, Iran

Table s1. Pollution index (PI) and NPI values of soil samples

FID	PI-Pb_	PI-Zn_	PI-Cu_	PI-As_	Average	Maximum	NPI
1	0.34	0.78	1.55	1.24	0.98	1.55	1.30
2	0.37	0.81	2.06	1.26	1.12	2.06	1.66
3	0.91	0.98	0.87	0.42	0.80	0.98	0.89
4	1.05	1.39	2.15	0.77	1.34	2.15	1.79
5	2.38	3.22	2.47	1.15	2.31	3.22	2.80
6	2.80	3.78	2.66	1.22	2.61	3.78	3.25
7	0.83	0.90	0.73	0.15	0.65	0.90	0.79
8	2.65	1.48	2.37	0.44	1.73	2.65	2.24
9	2.08	2.09	2.51	0.48	1.79	2.51	2.18
10	2.56	3.21	2.60	0.56	2.23	3.21	2.76
11	0.41	0.14	2.07	0.31	0.73	2.07	1.56
12	0.46	0.95	2.17	0.42	1.00	2.17	1.69
13	1.00	0.96	2.17	0.42	1.14	2.17	1.73
14	2.14	1.30	2.37	0.44	1.56	2.37	2.01
15	2.96	1.39	2.45	0.64	1.86	2.96	2.47
16	2.68	2.10	2.66	0.75	2.04	2.68	2.38
17	3.40	3.07	2.80	0.95	2.55	3.40	3.01
18	0.34	0.14	0.33	0.16	0.24	0.34	0.30
19	0.43	0.71	1.60	0.31	0.76	1.60	1.25
20	0.51	0.81	1.65	0.36	0.83	1.65	1.31
21	0.60	0.90	2.06	0.43	1.00	2.06	1.62
22	0.74	0.91	2.16	0.64	1.11	2.16	1.72

✉ Corresponding Author. E-mail: A.nazarpour@iauahvaz.ac.ir; Ahad.nazarpour@gmail.com, Phone: +989167729443, Fax: +986133771910

23	1.00	1.04	2.32	0.95	1.33	2.32	1.89
24	2.34	1.34	2.35	1.02	1.76	2.35	2.08
25	3.38	3.91	2.96	1.32	2.89	3.91	3.44
26	0.46	0.21	0.50	0.22	0.35	0.50	0.43
27	0.46	0.75	1.25	0.22	0.67	1.25	1.00
28	0.69	0.76	1.60	0.35	0.85	1.60	1.28
29	0.69	0.79	2.08	0.38	0.98	2.08	1.63
30	1.02	0.86	2.15	0.57	1.15	2.15	1.72
31	1.05	1.19	2.32	0.62	1.29	2.32	1.88
32	2.24	1.19	2.42	0.77	1.65	2.42	2.07
32	2.27	1.23	2.43	0.83	1.69	2.43	2.09
33	2.64	2.13	2.51	1.34	2.16	2.64	2.41
34	0.66	0.72	1.30	0.31	0.75	1.30	1.06
35	0.89	0.84	1.35	0.31	0.85	1.35	1.13
36	1.02	0.90	2.13	0.38	1.11	2.13	1.70
37	2.10	0.90	2.18	0.44	1.40	2.18	1.84
38	2.10	0.99	2.19	0.57	1.46	2.19	1.86
40	2.29	2.14	2.30	0.64	1.84	2.30	2.08
41	2.30	3.26	2.34	0.75	2.16	3.26	2.77
42	2.07	3.55	2.38	0.89	2.22	3.55	2.96
43	2.80	3.65	2.80	0.97	2.56	3.65	3.15
44	1.03	0.84	1.45	0.05	0.84	1.45	1.19
45	1.05	1.09	1.95	0.08	1.04	1.95	1.56
46	1.08	1.19	2.13	0.13	1.13	2.13	1.70
47	2.16	1.22	2.15	0.16	1.42	2.16	1.83
48	2.22	1.23	2.17	0.28	1.47	2.22	1.88
49	2.36	1.46	2.18	0.28	1.57	2.36	2.00
50	2.68	2.08	2.39	0.54	1.92	2.68	2.33
51	2.07	3.31	3.05	0.55	2.25	3.31	2.83
52	2.64	3.65	4.55	0.64	2.87	4.55	3.81
53	0.46	0.16	0.28	0.17	0.27	0.46	0.37
54	0.60	0.19	0.63	0.22	0.41	0.63	0.53
55	0.69	0.73	0.87	0.22	0.63	0.87	0.76
56	0.75	0.81	1.05	0.29	0.72	1.05	0.90
57	0.97	0.91	1.35	0.38	0.90	1.35	1.15
58	1.00	0.92	1.95	0.43	1.08	1.95	1.58
59	1.06	0.95	2.07	0.53	1.15	2.07	1.68
60	1.17	0.99	2.08	0.56	1.20	2.08	1.69
61	2.24	1.25	2.17	0.60	1.57	2.24	1.93
62	2.33	1.39	2.34	0.62	1.67	2.34	2.04
63	2.36	2.16	2.44	0.76	1.93	2.44	2.20
64	2.06	3.47	4.03	0.78	2.58	4.03	3.38
65	2.42	2.27	4.10	1.02	2.45	4.10	3.38

66	0.46	0.15	0.38	0.17	0.29	0.46	0.38
67	0.57	0.77	0.70	0.18	0.56	0.77	0.67
68	0.74	0.86	0.97	0.23	0.70	0.97	0.84
69	0.83	0.87	0.97	0.24	0.73	0.97	0.86
70	1.04	0.92	1.30	0.30	0.89	1.30	1.11
71	2.08	0.98	2.09	0.40	1.39	2.09	1.77
72	2.10	0.99	2.13	0.51	1.43	2.13	1.82
73	2.30	1.10	2.22	0.51	1.53	2.30	1.95
74	2.35	1.30	2.46	0.57	1.67	2.46	2.10
75	2.95	1.40	2.47	0.62	1.86	2.95	2.47
76	2.98	3.23	2.57	0.71	2.37	3.23	2.83
77	2.10	3.26	2.66	0.88	2.23	3.26	2.79
78	2.28	3.62	2.95	0.89	2.43	3.62	3.08
79	3.46	3.65	4.03	1.16	3.07	4.03	3.58
80	0.66	0.19	0.63	0.24	0.43	0.66	0.56
81	0.74	0.75	1.55	0.55	0.90	1.55	1.27
82	1.00	0.81	1.90	0.55	1.06	1.90	1.54
83	1.05	0.96	2.09	0.58	1.17	2.09	1.69
84	2.14	1.08	2.13	0.60	1.49	2.14	1.84
85	2.31	1.16	2.23	0.64	1.59	2.31	1.98
86	2.20	1.16	2.26	0.68	1.57	2.26	1.95
87	2.97	1.19	2.34	0.75	1.81	2.97	2.46
88	2.11	1.21	2.35	0.82	1.62	2.35	2.02
89	2.04	1.42	2.37	0.97	1.70	2.37	2.06
90	2.06	1.45	2.37	0.97	1.71	2.37	2.07
91	2.16	2.08	2.66	1.02	1.98	2.66	2.34
92	2.16	3.20	2.80	1.36	2.38	3.20	2.82
93	3.15	2.66	2.97	1.48	2.57	3.15	2.87
94	0.40	0.19	0.53	0.18	0.32	0.53	0.44
95	0.60	0.78	0.53	0.22	0.53	0.78	0.67
96	0.60	0.92	0.70	0.33	0.64	0.92	0.79
97	1.08	0.99	1.35	0.35	0.94	1.35	1.16
98	2.10	0.99	2.11	0.38	1.39	2.11	1.79
99	2.11	1.10	2.11	0.42	1.44	2.11	1.81
100	2.11	1.37	2.15	0.44	1.52	2.15	1.86
101	2.24	1.49	2.18	0.49	1.60	2.24	1.95
102	2.90	2.01	2.19	0.74	1.96	2.90	2.47
103	2.67	2.01	2.23	0.75	1.91	2.67	2.32
104	2.06	2.01	2.25	0.75	1.77	2.25	2.03
105	2.12	2.11	2.26	0.77	1.81	2.26	2.05
106	2.16	3.18	2.28	0.95	2.14	3.18	2.71
107	2.18	3.25	2.58	0.97	2.25	3.25	2.79
108	2.19	3.57	2.66	0.97	2.35	3.57	3.02

109	2.24	3.72	2.66	1.05	2.42	3.72	3.14
110	2.28	3.76	4.09	1.23	2.84	4.09	3.52
111	2.48	3.21	4.11	1.27	2.77	4.11	3.50
112	0.34	0.81	0.37	0.09	0.40	0.81	0.64
113	0.54	0.84	0.40	0.21	0.50	0.84	0.69
114	0.74	0.96	1.05	0.28	0.76	1.05	0.92
115	2.08	0.98	1.63	0.31	1.25	2.08	1.72
116	2.18	1.26	2.07	0.50	1.50	2.18	1.87
117	2.17	1.26	2.12	0.57	1.53	2.17	1.88
118	2.22	1.31	2.13	0.61	1.57	2.22	1.92
119	2.04	1.45	2.15	0.63	1.57	2.15	1.89
120	2.18	2.03	2.38	0.64	1.81	2.38	2.11
121	2.47	3.21	2.91	0.69	2.32	3.21	2.80
122	2.48	3.50	3.03	0.75	2.44	3.50	3.02
123	2.56	3.83	3.04	0.75	2.54	3.83	3.25
124	2.72	3.88	4.49	0.77	2.97	4.49	3.80
125	3.54	3.22	4.55	0.83	3.04	4.55	3.87
126	4.17	3.32	5.34	0.95	3.45	5.34	4.50
127	0.34	0.78	0.90	0.11	0.53	0.90	0.74
128	0.54	0.82	2.06	0.28	0.93	2.06	1.60
129	1.03	0.82	2.12	0.33	1.07	2.12	1.68
130	1.07	2.07	2.16	0.44	1.43	2.16	1.83
131	2.33	2.08	2.30	0.44	1.79	2.33	2.08
132	2.36	3.18	2.54	0.75	2.21	3.18	2.74
133	2.25	3.48	2.66	0.82	2.30	3.48	2.95
134	2.54	3.79	4.03	1.01	2.84	4.03	3.48
135	2.68	3.93	4.15	1.02	2.95	4.15	3.60
136	2.92	3.13	4.27	1.05	2.84	4.27	3.62
137	3.60	3.15	4.41	1.12	3.07	4.41	3.80
138	3.98	3.27	4.69	1.62	3.39	4.69	4.09
139	0.34	0.81	0.57	0.16	0.47	0.81	0.66
140	1.05	1.25	2.13	0.21	1.16	2.13	1.71
141	2.20	1.49	2.45	0.58	1.68	2.45	2.10
142	2.10	2.11	2.47	0.74	1.85	2.47	2.18
143	2.69	3.46	2.58	0.75	2.37	3.46	2.97
144	2.65	3.50	2.58	0.75	2.37	3.50	2.99
145	2.34	3.52	2.58	0.75	2.30	3.52	2.97
146	2.69	3.53	2.66	0.81	2.42	3.53	3.03
147	2.12	3.65	2.74	0.97	2.37	3.65	3.08
148	2.44	3.65	2.92	0.97	2.50	3.65	3.13
149	2.96	3.86	3.03	1.14	2.75	3.86	3.35
150	0.46	0.18	0.97	0.10	0.43	0.97	0.75
151	0.66	0.83	2.11	0.14	0.93	2.11	1.63

152	1.00	0.91	2.12	0.15	1.04	2.12	1.67
153	2.13	1.09	2.17	0.22	1.40	2.17	1.83
154	2.22	1.45	2.18	0.44	1.57	2.22	1.92
155	2.22	1.48	2.21	0.51	1.60	2.22	1.94
156	2.23	1.75	2.22	0.55	1.69	2.23	1.98
157	2.30	2.01	2.31	0.77	1.85	2.31	2.09
158	2.67	2.18	2.56	0.81	2.06	2.67	2.38
159	2.54	3.21	2.73	0.84	2.33	3.21	2.80
160	2.25	3.55	2.80	1.06	2.41	3.55	3.03
161	2.44	3.57	4.25	1.14	2.85	4.25	3.61
162	0.83	0.93	2.09	0.08	0.98	2.09	1.63
163	1.03	1.22	2.12	0.11	1.12	2.12	1.69
164	1.06	1.22	2.18	0.17	1.16	2.18	1.75
165	1.06	1.45	2.19	0.17	1.22	2.19	1.77
166	2.11	1.46	2.22	0.31	1.53	2.22	1.90
167	2.17	1.49	2.29	0.35	1.57	2.29	1.96
168	2.33	2.03	2.38	0.55	1.82	2.38	2.12
169	2.04	3.21	2.67	0.62	2.13	3.21	2.72
170	2.05	3.53	2.70	0.91	2.30	3.53	2.98
171	2.14	3.63	2.93	0.95	2.41	3.63	3.09
172	2.28	3.67	3.02	2.20	2.79	3.67	3.26
173	0.74	0.90	2.15	0.11	0.97	2.15	1.67
174	2.09	1.16	2.15	0.11	1.38	2.15	1.81
175	2.15	1.31	2.19	0.22	1.47	2.19	1.86
176	2.17	1.39	2.22	0.42	1.55	2.22	1.91
177	2.18	1.48	2.23	0.51	1.60	2.23	1.94
178	2.19	1.75	2.34	0.51	1.70	2.34	2.04
179	2.20	2.01	2.39	0.62	1.80	2.39	2.12
180	2.21	2.03	2.47	0.74	1.86	2.47	2.19
181	2.30	2.18	2.70	0.83	2.00	2.70	2.38
182	2.34	3.19	2.71	0.96	2.30	3.19	2.78
183	2.16	2.38	2.95	1.02	2.13	2.95	2.57
184	0.43	0.78	1.30	0.09	0.65	1.30	1.03
185	0.83	0.84	2.08	0.17	0.98	2.08	1.63
186	0.89	0.98	2.13	0.31	1.08	2.13	1.69
187	2.10	0.99	2.23	0.50	1.46	2.23	1.88
188	2.13	1.19	2.38	0.69	1.60	2.38	2.03
189	2.54	1.75	2.41	0.75	1.86	2.54	2.23
190	2.78	2.07	2.71	0.75	2.08	2.78	2.45
191	2.68	3.21	2.74	0.77	2.35	3.21	2.81
192	2.44	3.27	2.83	0.88	2.35	3.27	2.85
193	2.87	3.30	4.60	0.93	2.93	4.60	3.86
194	3.96	3.95	4.69	1.42	3.50	4.69	4.14

195	0.31	0.76	0.93	0.17	0.54	0.93	0.76
196	0.89	0.77	1.85	0.24	0.94	1.85	1.47
197	1.00	0.84	1.95	0.35	1.03	1.95	1.56
198	2.13	1.12	2.11	0.36	1.43	2.13	1.81
199	2.17	3.22	2.18	0.41	1.99	3.22	2.68
200	2.23	3.25	2.22	0.46	2.04	3.25	2.72
202	2.07	3.26	2.34	0.51	2.04	3.26	2.72
203	2.12	3.26	2.68	0.71	2.19	3.26	2.78
204	2.84	3.34	2.93	0.77	2.47	3.34	2.94
205	3.69	3.62	4.37	0.84	3.13	4.37	3.80
206	0.34	3.02	5.89	0.88	2.53	5.89	4.53
207	1.06	0.77	0.37	0.21	0.60	1.06	0.86
208	2.16	0.99	2.12	0.24	1.38	2.16	1.81
209	2.33	1.17	2.21	0.28	1.50	2.33	1.96
210	2.14	1.25	2.29	0.58	1.56	2.29	1.96
211	2.30	1.45	2.45	0.71	1.73	2.45	2.12
212	2.36	3.50	2.48	0.97	2.33	3.50	2.98
213	2.44	3.62	4.03	1.02	2.78	4.03	3.46
214	2.85	3.73	4.26	1.14	3.00	4.26	3.68
215	2.26	3.07	4.41	1.42	2.79	4.41	3.69
216	2.40	1.25	2.15	0.11	1.48	2.40	1.99
217	2.44	1.50	2.40	0.31	1.66	2.44	2.09
218	2.51	2.18	2.59	0.42	1.92	2.59	2.28
219	2.84	3.21	2.79	0.75	2.40	3.21	2.83
220	2.18	3.91	4.46	0.95	2.88	4.46	3.75
221	2.52	2.12	2.63	0.22	1.87	2.63	2.28
222	2.68	3.30	2.63	0.28	2.22	3.30	2.82
223	2.68	3.36	2.74	0.38	2.29	3.36	2.87
224	3.24	3.62	2.89	0.57	2.58	3.62	3.14
225	2.04	3.86	4.65	0.95	2.88	4.65	3.87
226	2.46	3.28	2.49	0.22	2.11	3.28	2.76
227	2.47	3.48	2.60	0.45	2.25	3.48	2.93

Table S2. Pb isotopic ratios in selected samples

Sample	$^{207}Pb/^{206}Pb$	$^{208}Pb/^{206}Pb$
AH-S14	0.92	2.125
AH-S14	0.86	2.113
AH-S37	0.94	2.126
AH-S39	0.93	2.155
AH-S43	0.88	2.128
AH-S76	0.89	2.123
AH-S161	0.93	2.142
AH-S168	0.91	2.1296
AH-S183	0.88	2.133
AH-S187	0.889	2.123
AH-S193	0.871	2.1424
AH-S209	0.94	2.131
AH-S211	0.96	2.127
AH-S216	0.85	2.129
AH-S27	0.82	2.121
AH-background 1	0.69	2.084
AH-background 2	0.71	2.086
AH-background 3	0.67	2.091
AH-background 4	0.68	2.093
Khuzestan Dust PM 2.5	0.861	2.11
Khuzestan Dust PM 10	0.875	2.134
Khuzestan Dust TSP	0.848	2.12
Paint (yellow line on road)	0.84	2.115
Battery	0.863	2.11
Broken Hill	0.95	2.12
Mississippi Vally	0.82	2.093
Shimanto Shales	0.847	2.102
Vehicle exhust	0.884	2.142
Vehicle exhust	0.891	2.148
Vehicle exhust	0.895	2.15
Vehicle exhust (leaded gasoline)	1.11	2.175
Vehicle exhust (leaded gasoline)	1.12	2.189
Vehicle exhust (leaded gasoline)	1.1	2.185
Vehicle exhust (leaded gasoline)	1.13	2.192
Vehicle exhust (leaded gasoline)	1.17	2.18
Vehicle exhust (unleaded gasoline)	0.875	2.127
Vehicle exhust (unleaded gasoline)	0.879	2.132
Vehicle exhust (unleaded gasoline)	0.873	2.129
Vehicle exhust (unleaded gasoline)	0.876	2.136

Vehicle exhust (unleaded gasoline)	0.872	2.128
chemical fertilizer	0.871	2.1
industry waste	0.874	2.11

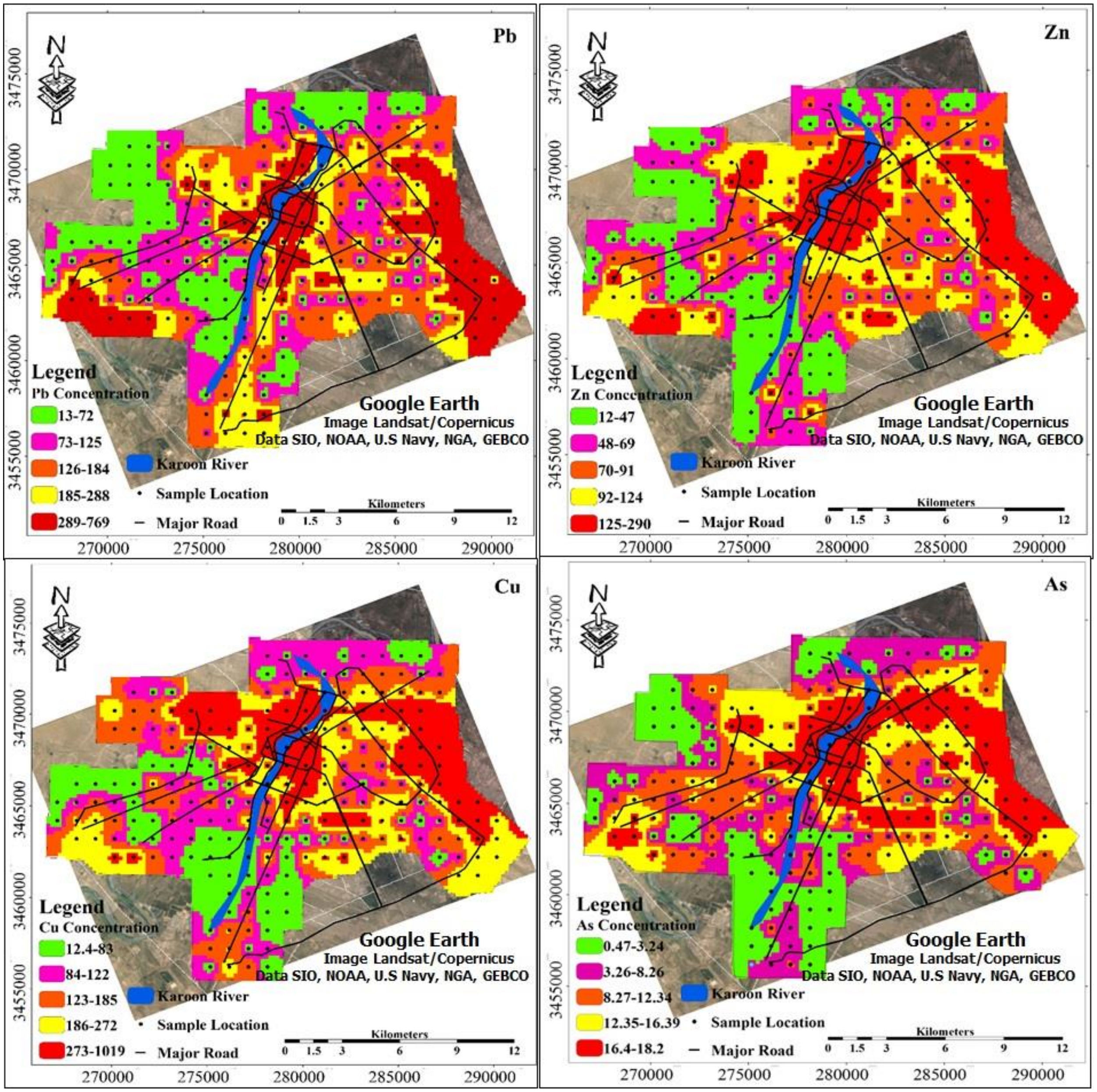

Fig 1. Spatial distribution of studied toxic metals in Ahvaz surface soil samples, the image was made by ArcGIS10.2, background from Google Earth (Image: Google, Landsat/Copernicus).

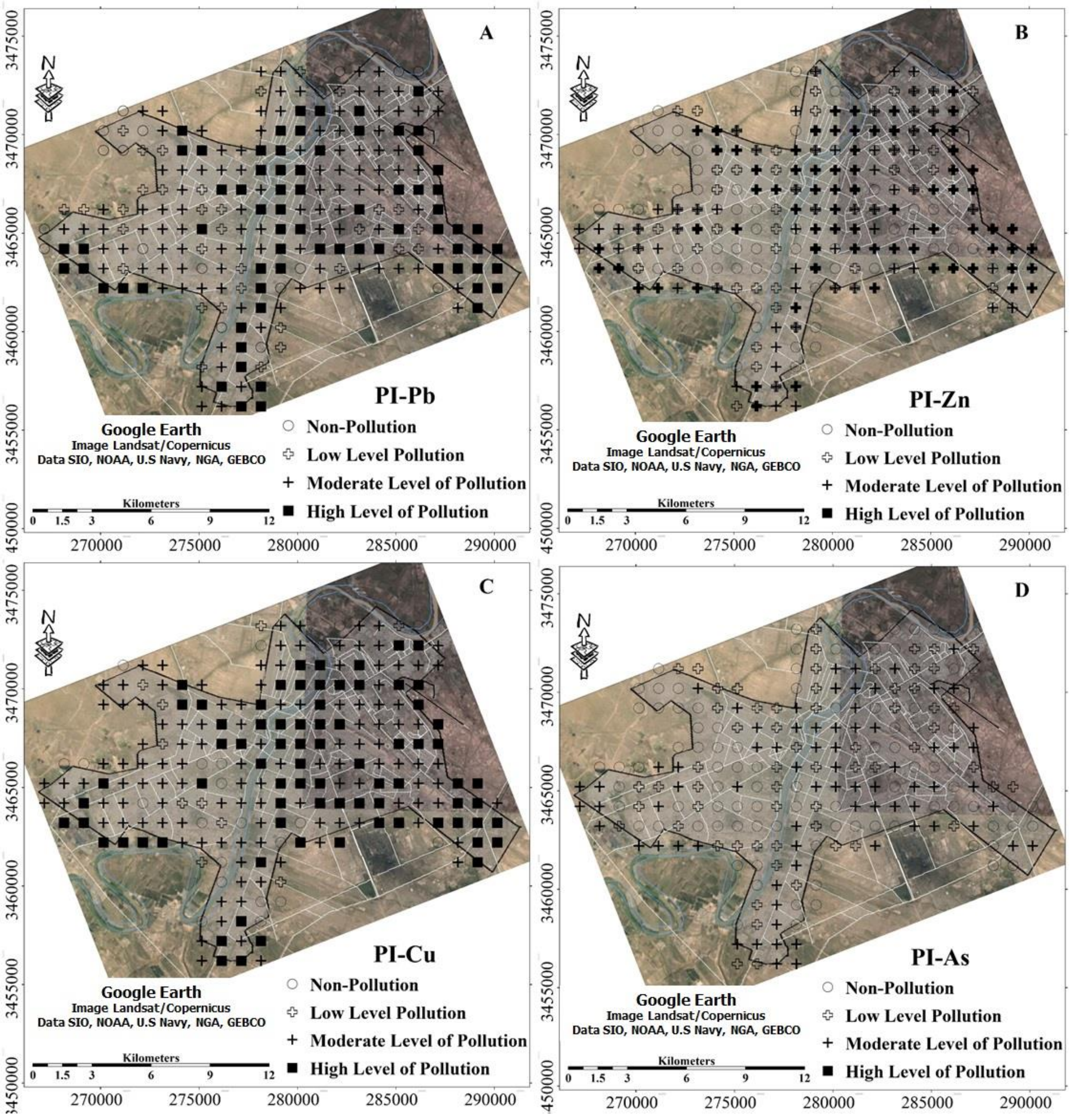

Fig 2. Spatial distribution of pollution index (PI) of studied toxic metals in Ahvaz surface soil samples, the image was made by ArcGIS10.2, background from Google Earth (Image: Google, Landsat/Copernicus).
